# Locating influential nodes in complex networks

**DOI:** 10.1038/srep19307

**Published:** 2016-01-18

**Authors:** Fragkiskos D. Malliaros, Maria-Evgenia G. Rossi, Michalis Vazirgiannis

**Affiliations:** 1 Computer Science Laboratory, École Polytechnique, 91120 Palaiseau, France

## Abstract

Understanding and controlling spreading processes in networks is an important topic with many diverse applications, including information dissemination, disease propagation and viral marketing. It is of crucial importance to identify which entities act as influential spreaders that can propagate information to a large portion of the network, in order to ensure efficient information diffusion, optimize available resources or even control the spreading. In this work, we capitalize on the properties of the *K*-truss decomposition, a triangle-based extension of the core decomposition of graphs, to locate individual influential nodes. Our analysis on real networks indicates that the nodes belonging to the maximal *K*-truss subgraph show better spreading behavior compared to previously used importance criteria, including node degree and *k*-core index, leading to faster and wider epidemic spreading. We further show that nodes belonging to such dense subgraphs, dominate the small set of nodes that achieve the optimal spreading in the network.

Spreading processes in complex networks have gained great attention from the research community due to the plethora of applications that they occur, ranging from the spread of news and ideas to the diffusion of influence and social movements and from the outbreak of a disease to the promotion of commercial products. Being able to understand the underlying mechanisms that govern such processes is a crucial task with direct applications in various fields, including epidemiology, collective dynamics and viral marketing.

Typically, the interactions among individuals are responsible for the formation of information pathways in the network and to this extend, their position and topological properties have direct effect to the spreading phenomena occurring in the network. That way, a fundamental aspect on understanding and controlling the spreading dynamics is the identification of influential spreaders that can diffuse information to a large portion of the network. For example, in the case of virus propagation, such as influenza, the transmission of the disease mainly depends on the extend of contacts of the infected person to the susceptible population; thus, being able to locate and vaccinate individuals with good spreading properties can prevent from a potential outbreak of the disease, leading to efficient strategies of epidemic control. In a similar way, suppose that our goal is to promote an idea or a product in order to be adopted by a large fraction of individuals in the network. A key idea behind viral marketing is the word-of-mouth effect^1^; individuals that have already adopted the product, recommend it to their friends who in turn do the same to their own social circle, forming a cascade of recommendations^2^. The basic question here is how to target a few initial individuals (e.g., by giving them free samples of the product or explaining them the idea), that can maximize the spread of influence in the network, leading to a successful promotion campaign.

The problem of identifying nodes with good spreading properties in networks, can be further split in two subtopics: (i) identification of individual influential nodes and (ii) identification of a group of nodes that, by acting all together, are able to maximize the total spread of influence. In this work, we focus on the problem of identifying single influential spreaders in networks. A straightforward approach towards finding effective spreading predictors, is to consider node centrality criteria and in particular the one of degree centrality. In fact, several studies have examined how the existence of heavy-tailed degree distribution in real-world networks^3^,^4^,^5^ is related to cascading effects concerning the robustness of such complex systems^4^,^6^,^7^,^8^. Nevertheless, there exist cases where a node can have arbitrarily high degree, while its neighbors are not well-connected, making degree a not very accurate predictor of the spreading properties. For example, this can occur when a high degree node is located to the periphery of the network. In fact, the spreading properties of a node are strongly related to the ones of its neighbors in the graph, and thus, global centrality criteria seem to be more appropriate for this task.

Towards this direction, several approaches have been proposed in the related literature. Lu *et al*.^9^ proposed LeaderRank, a random walk-based algorithm similar to PageRank^10^ for identifying influential users in social networks. Later, Li *et al*.^11^ extended LeaderRank to properly detect influential nodes in weighted networks. Chen *et al*.^12^ proposed a semi-local centrality measure which serves as a trade-off between degree and other computationally complex measures (betweenness and closeness centrality). Additionally, Chen *et al*.^13^ proposed ClusterRank, a local ranking method that takes into account the clustering coefficient of a node while in another approach^14^, the diversity of the paths that emanate from a node was considered. The main idea was that the spreading ability of a node may be reduced if its propagation depends only on a few paths, while the rest ones lead to dead ends.

Of particular importance is the work by Kitsak *et al*.^15^, which stressed out that highly connected nodes or those having high betweenness and closeness centralities, have little effect on the range of the spreading process. The main finding of their work was that, less connected but strategically placed nodes in the core of the network, are able to disseminate information to a larger part of the population. To quantify the core-periphery structure of networks, they applied the *k*-core decomposition algorithm^16^,^17^,^18^—a pruning process that removes nodes which do not satisfy a particular degree-based threshold. Their results indicated that nodes belonging to the maximal *k*-core subgraph are able to infect a larger portion of the network, compared to node degree or betweenness centrality, making the *k*-core number of a node a more accurate spreading predictor. Furthermore, extracting the *k*-core subgraph is a more efficient task compared to the heavy computation required by some centrality criteria (e.g., betweenness). Nevertheless, the resolution of *k*-core decomposition is quite coarse; depending on the structure of the network, many nodes will be assigned the same *k*-core number at the end of the process, even if their spreading capability differs from each other. Furthermore, building upon the good performance of the *k*-core decomposition, several extensions have been proposed^19^,^20^,^21^,^22^,^23^,^24^,^25^ (see Supplementary Note 6).

Our proposed approach moves on a similar axis as the one by Kitsak *et al*.^15^; we argue that the topological properties of the nodes play a crucial role towards understanding their spreading capabilities. In particular, we consider that only a relatively small fraction of the nodes extracted by the *k*-core decomposition method corresponds to highly influential nodes. To that end, we propose the *K*-truss decomposition of a graph^26^,^27^,^28^, a triangle-based extension of the *k*-core decomposition, as a more accurate method to identify privileged spreaders. The algorithm is able to extract a more refined and even more dense subgraph of the initial graph–compared to the *k*-core decomposition–as the *K*-truss is structurally more close to a clique. In fact, the *K*-truss subgraph corresponds to the *core* of a *k*-core that filters out less important information. We perform experiments on large scale real-world networks, showing that the nodes belonging to the maximal *K*-truss subgraph of the network show better spreading behavior under the SIR epidemic model–compared to previously used importance criteria–leading to faster and wider epidemic spreading. Furthermore, the extracted nodes dominate the small set of nodes that achieve the optimal spreading in the network.

## Results

Let 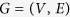 be an undirected graph with 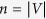 nodes and 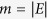 edges. In graph theory, the *K*-truss subgraph 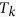 of a graph *G*, is defined as the largest subgraph where all edges belong to at least 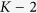 triangles, i.e., cycle subgraphs of length three^26^,^27^. Respectively, an edge 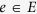 has truss number 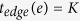 if it belongs to 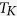 but not to 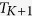. Let 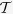 denotes the set of nodes belonging to the maximal *K*-truss subgraph of the graph. In this article, we argue that this set contains highly influential nodes with good spreading properties. It has been shown that the maximal *k*-core and *K*-truss subgraphs (i.e., maximum values for *k, K*) overlap, with the latter being a subgraph of the former; the *K*-truss subgraph represents the most connected part of the corresponding *k*-core, leading to a significant reduction of the set of nodes with respect to their structural properties and position within the graph (see Supplementary Note 3). Building upon the fact that the nodes belonging to the maximal *k*-core of the graph have good spreading properties^15^, here we further refine this set of the most influential nodes, showing that the nodes belonging to set 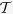 defined above perform even better, leading to faster and wider epidemic spreading.

We study real-world networks arising from online social networking and communication platforms (all datasets are publicly available^29^). In particular, we investigate the following network datasets: (i) EMAIL-ENRON and (ii) EMAIL-EUALL, two email communication networks; (iii) EPINIONS which is an online social network created from the product review website Epinions.com; (iv) WIKI-VOTE, a network created by all the voting data between administrators of Wikipedia; (v) WIKI-TALK, created by the interaction data between Wikipedia users; (vi) SLASHDOT, which is created by the friendship relationships in the technology review website Slashdot.org. All datasets are considered undirected and unweighted; also, the largest connected component was used in the experiments. High level characteristics of the networks are shown in Table 1 (see Supplementary Note 1 for more details about the datasets).

Before presenting the results about the spreading properties, we examine the maximum level of the *K*-truss decomposition, i.e., value 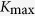, for the various graphs. As we can observe from Table 1, 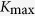 values vary from dataset to dataset, but compared to the 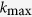 values of the *k*-core decomposition, they tend to be much smaller. This is rather expected since the *K*-truss decomposition relies on triangle participation, which is a more strict criterion compared to node degree. This last point is also a justification for the differences on the number of nodes belonging to the truss set 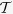 and core set 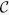 (i.e., the set of nodes belonging to the maximal k-core subgraph of the graph - see Methods for more details). Although these sets are overlapping, the one that corresponds to *K*-truss has significantly smaller size compared to the maximal *k*-core subgraph. This was also one of the motivations of the proposed work; since the nodes of the maximal *k*-core subgraph perform well in information spreading, how to further refine this set by selecting a small subset that is characterized by even better spreading properties.

### Evaluating the spreading performance

In the experimental results that follow, we are comparing the spreading performance of the nodes belonging to the set 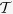 (**truss** method), to those belonging to the set 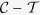 (**core** method), i.e., the nodes belonging to the maximal *k*-core excluding those that belong to the maximal *K*-truss of the graph–since 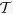 is subset of 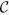, as discussed above. The **core** method constitutes the basic baseline approach, since it has been shown that outperforms other well known node importance criteria such as betweenness centrality^15^. For completeness in the experimental evaluation, we also compare the spreading capabilities of the nodes that belong to the maximal *K*-truss subgraph to those belonging to the set 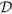 that contains the highest degree nodes in the graph (**top degree** method); we choose 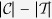 high degree nodes to achieve fair comparison between the different methods.

To study the spreading process and evaluate the performance of the nodes extracted by the *K*-truss decomposition method, we apply the SIR epidemic model^30^,^31^. Initially, we set one node to be in the infected state *I*. This node corresponds to our single spreader, that is chosen by the *K*-truss decomposition method (in general, the initial node can be any node of the graph; the same procedure is also performed for the baseline methods). The rest of the nodes are assigned to the susceptible state *S*. At each time step, the infected nodes can infect their susceptible neighbors with probability *β* (i.e., infection rate). Furthermore, the nodes that have been previously infected can recover from the disease with probability *γ* (i.e., recovery rate). The process is repeated until no more new nodes get infected. Let 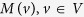 be the size of the population that is infected by the epidemic triggered by node *v* (average value over multiple executions of the model - see also Supplementary Note 4 for a more detailed description about the simulation of the spreading process). Setting high *β* values, a relatively large fraction of the nodes will be infected and thus the role of individual nodes in the spreading process is diminished. In our approach, we set *β* close to the epidemic threshold 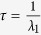, where 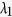 is the largest eigenvalue of the adjacency matrix of the network^32^. We also set parameter 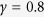, as used by Kitsak *et al*.^15^. As we will present later, we have performed experiments with several values of *β* and *γ* and the results are persistent concerning the comparison of the proposed method to other baselines.

To evaluate the spreading efficiency of the methods, we focus on the following quantities: (i) the number of nodes that become infected at each time step of the process and the corresponding cumulative one; (ii) the total number of infected nodes at the end of the epidemic; (iii) the time step where the epidemic fades out. For each node, we repeat the simulation 100 times (10 times for the WIKI-TALK graph due to its large size) and report the average behavior. In each case, we repeat the above for all the respective nodes and calculate the average behavior for the nodes of each set (**truss** method versus the two baselines **core** and **top degree**). The experimental results are shown in Table 2. The values of parameter *β* of the SIR model for each graph, are shown in Table 1. Table 2 shows the number of the newly infected nodes for some of the first ten time steps of the spreading process, which we consider as the outbreak of the epidemic (see Supplementary Table 1 for an extended version of this table including the number of infected nodes for all the first ten steps of the process; also Supplementary Table 2 shows the cumulative number of infected nodes per step). We also report the total number of nodes that were infected at the end of the process (*Final step*) and the time step where the epidemic dies out (*Max step*).

As we can observe, the **truss** method achieves significantly higher infection rate during the first steps of the epidemic. Furthermore, in almost all cases, the total number of infected nodes at the end of the process (*Final step*) is larger, while the fade out occurs earlier (*Max step*). Lastly, as we discussed above, the number of nodes in the truss set 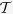 is much smaller compared to the set 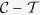 (Table 1). By refining significantly the set of influential nodes in truss set 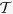, the “weaker” spreaders of 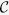 are left in core set 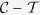, explaining the inferior behavior of the **core** method compared to **top degree**. Some small deviations from this behavior are observed in the SLASHDOT and WIKI-TALK graphs. In the SLASHDOT graph, the best performance is achieved by the **top degree** method, which from the very first steps is able to infect a larger amount of nodes. In the case of the WIKI-TALK graph, although the total number of infected nodes at the end (*Final step*) of the epidemic is almost the same for all methods, the proposed **truss** method performs quite effectively during the first steps of the process. In fact, it significantly outperforms both baseline methods achieving an increase of almost 23% on the cumulative number of infected nodes compared to both **core** and **top degree** methods, at the sixth step of the process.

We have also computed the cumulative difference of the number of infected nodes per step achieved by the methods. Let 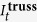 be the number of infected nodes at step *t* achieved by the **truss** method (similar for **core** and **top degree**). We define the cumulative difference for the **truss** and **core** methods at step *t* as


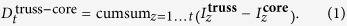


**Figure 1:**
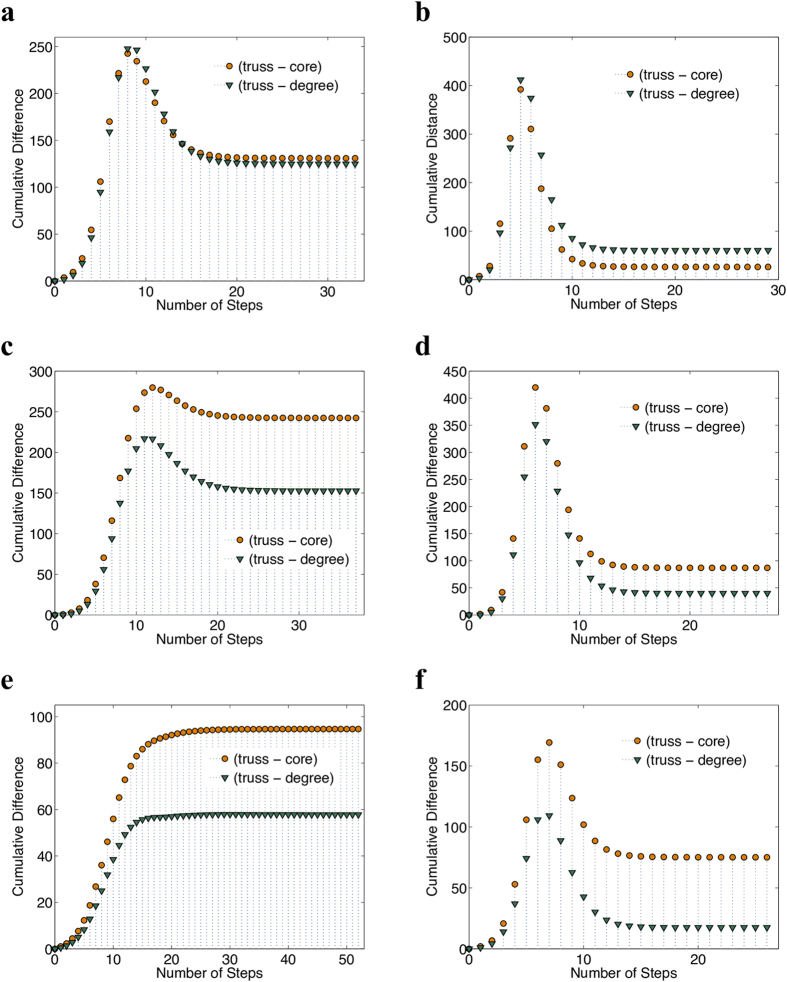
Comparative performance of the proposed truss method versus the core and top degree methods. We show results for the following networks and the corresponding infection rates *β*: Email-Enron (**a**) 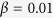, **(b)** 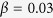; Epinions **(c)** 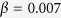, (**d**) 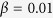; Wiki-Vote (**e**) 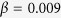, (**f**) 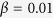. Each plot depicts the cumulative difference of the infected nodes per step achieved by the **truss** method vs. the **core** (truss - core) and **top degree** (truss - degree) methods. Parameter *γ* of the SIR models is set to 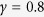. In all cases, the proposed **truss** method outperforms the baselines, leading to more effective information spreading.

Similarly, we can define the same quantity for the **truss** vs. **top degree** methods. The results for the EMAIL-ENRON, EPINIONS and WIKI-VOTE graphs are shown in Fig. 1 (see Supplementary Fig. 1 for the results of the EMAIL-EUALL and WIKI-TALK graphs). For each graph, we have performed experiments for two values of parameter *β* and 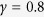. We observe that the cumulative difference of the number of nodes that are being infected at every step is always larger between **truss** and **core** than between **truss** and **top degree**. Both differences increase during the outbreak of the epidemic until they stabilize to the number of nodes which is actually the final difference of the number of nodes that got infected (i.e., entered state *I* of the SIR model) during the epidemic process of the two compared methods. Clearly, as in almost all cases the differences are always above zero, one can conclude to the effectiveness of information diffusion when the spreading is triggered by the nodes that belong to the maximal *K*-truss subgraph.

### Comparison to the optimal spreading

**Figure 2:**
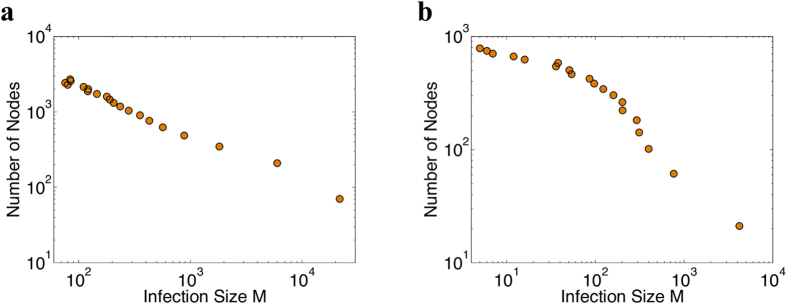
Spreading distribution of the nodes in the network, in log-log scale. We provide results for the following networks: **(a)** Email-Enron and **(b)** Wiki-Vote. The horizontal axis corresponds to the infection size *M* achieved by each node in the graph, after a binning process. The vertical axis captures the number of nodes that fall on each bin. Observe that only a small percentage of nodes achieves high spreading. In both cases, we have set 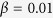 in the SIR model.

Since we lack ground-truth information about the best spreaders in the network, to further study the performance of the proposed *K*-truss decomposition method, we have examined the spreading achieved by each node of the graph. More precisely, we set each node 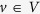 at the infected state *I* and simulate the spreading capabilities of this node using the SIR model, as described earlier. Figure 2 depicts the distribution of the nodes with respect to the infection size *M*, for the EMAIL-ENRON and WIKI-VOTE graphs (parameter *β* of the SIR model was set to 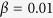 for this experiment). In both cases, the axes of the plot have been set to logarithmic scale. As we can observe, the distribution of the infection size *M* is skewed; only a small percentage of nodes are highly influential, while the majority of the nodes are able to infect only a small portion of the graph (small values of infection size *M*). Thus, our goal is to examine how the nodes detected by the *K*-truss decomposition are distributed on this small subset of spreading-efficient nodes. Note that, similar observations have been made for the rest of the graphs described at Table 1.

To that end, we rank the nodes 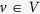 of the graph, according to the infection size 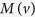. Let


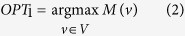


be the node that achieves that highest infection size 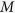 among all nodes in the graph, i.e., 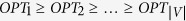. In order to examine how the nodes detected by the *K*-truss decomposition are distributed among the most efficient (optimal) spreaders, we consider a variable size window *W* over the ranked nodes and define 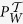 to be the fraction of nodes of set 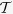 that can be found within *W* as follows:


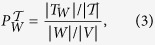


where *T*_*W*_ is the set of nodes 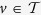 that are located in the window *W* of size 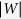 (in a similar way, we can define 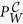 for the nodes of the maximal *k*-core subgraph). We are interested in examining how the quantities 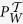 and 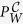 behave with respect to the size of the window *W*.

**Figure 3:**
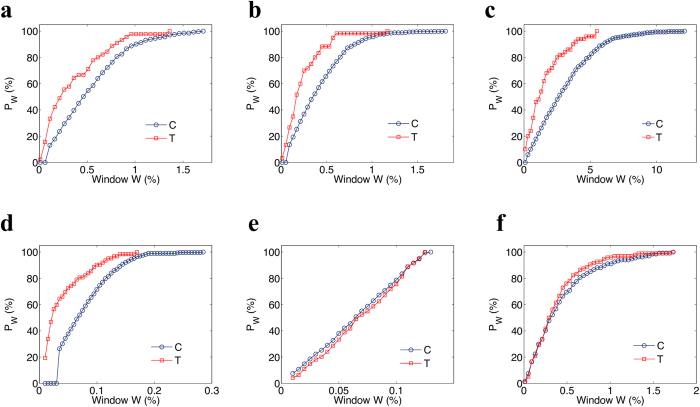
Ability of truss and core methods to identify the most efficient spreaders in the networks. We report results for the following networks: **(a)** Email-Enron, **(b)** Epinions, **(c)** Wiki-Vote, **(d)** Email-EuAll, **(e)** Wiki-Talk and **(f)** Slashdot. Distribution of the top-truss 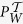 and top-core 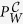 nodes among the nodes with optimal spreading properties under a window of size *W*. Observe that for small values of window size *W* (i.e., closer to the optimal spreading), the number of top-truss nodes is always higher compared to the number of top-core nodes.

Figure 3 depicts the distribution of the top-truss 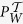 and top-core 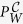 nodes, for various sizes of window *W* (i.e., fractions of the most efficient spreaders). As we can observe, for almost all datasets, 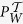 reaches the maximum value (i.e., 100%) relatively early and for small window sizes, compared to 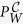. The maximum value of 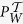 indicates that we have found all the nodes belonging to set 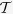 in the window of fractional size *W*. An early and intense upward trend of the curve implies that a large fraction of the nodes belonging to the set of interest 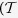 or 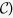, corresponds to nodes with the best spreading properties on the graph. For example, in the EMAIL-EUALL graph, the maximum of the nodes of set 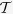 is reached in window 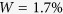, while in the case of set 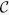 in window 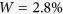. Thus, the nodes detected by the *K*-truss decomposition method (set 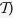 are better distributed among the most efficient spreaders, compared to those located by the *k*-core decomposition (set 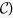. A slightly different behavior is observed in the WIKI-TALK and SLASHDOT graphs; in both graphs, the values of 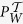 and 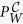 are very close to each other for almost all choices of window *W*, indicating that both sets have almost the same overlap with the set of optimal spreaders. Nevertheless, as we have already presented in Table 2, for those two datasets the spreading performance of the truss nodes achieved during the first steps of the epidemic is much better.

Furthermore, we are interested to study the distribution of the nodes’ truss number 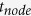 with respect to window *W*. Similar to what described above, we consider a fraction of the best spreaders in the graph (as specified by *W*) and we examine the distribution of all truss numbers (and not only the maximum one) within it. Since nodes with high truss number are of particular importance here, we have considered groups of nodes as follows: (i) individual groups for each of the top five truss numbers, i.e., 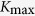 to 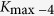. That way, the first group contains nodes with truss number equal to 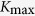, the second group of nodes with truss number 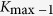 and so on. (ii) The rest of the groups concern truss numbers in the range 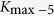 to 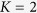, grouping together five consecutive truss numbers each time. For example, the sixth group contains nodes with truss number in the range 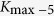 to 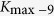. Note that, the last group may contain less than five truss numbers.

**Figure 4:**
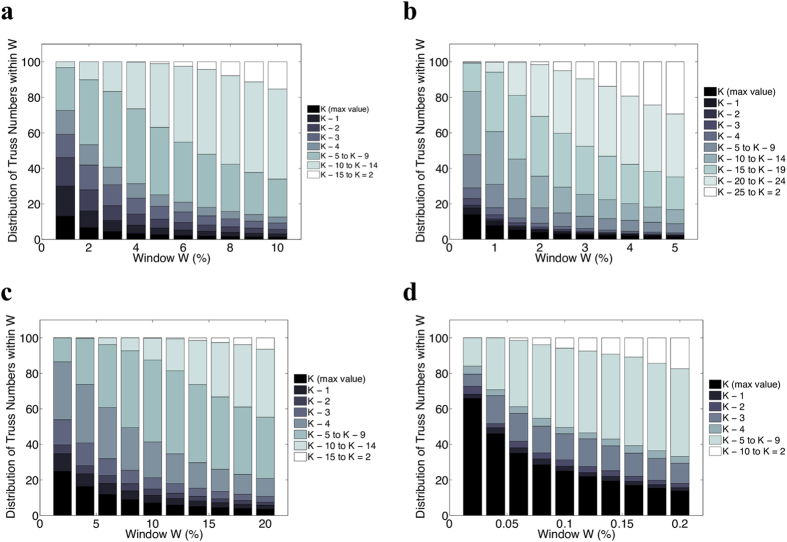
Distribution of node’s truss number with respect to the ranking of the nodes under their spreading effectiveness. We report results for the following datasets: **(a)** Email-Enron, **(b)** Epinions, **(c)** Wiki-Vote and **(d)** Email-EuAll. The nodes are classified in groups (different colors) depending on their truss number; for each window size *W*, we plot the distribution of truss numbers observed within it. Observe that, for small window sizes a relatively large number of the nodes belong to the first group, i.e., their truss number is 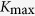. When the window is enlarged, the groups of lower truss numbers involve a large percentage of the considered nodes.

Figure 4 depicts the distribution of truss numbers for various values of window *W*. The colors on each bar correspond to the groups of truss number (darker colors for truss numbers closer to the maximum one). As we can observe in most of the datasets, for small values of window *W*, a large number of the nodes belong to the first group, i.e., their truss number is the maximum one. Since in most of the cases only a tiny fraction of the nodes of the graph belong to the very first groups (i.e., close to 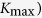, even for small window sizes we also observe nodes from groups that correspond to smaller truss numbers. As the window *W* increases, i.e., deviate from the optimal spreading behavior, groups of smaller truss numbers start to evolve. From these results, it is evident that the truss number is related to the spreading capabilities of the nodes. Until now, we had only examined the effect of the nodes that belong to the maximal *K*-truss subgraph. However, from this experiment we can conclude that, in general, nodes with high truss number tend to have good spreading properties–with the truss number being highly related to the spreading effect.

### Impact of infection and recovery rate on the spreading process

We have also examined the impact of the infection and recovery rate of the SIR model on the epidemic spreading achieved by the proposed method (**truss**) and the two baseline methods (**core** and **top degree**). To that end, we have simulated the spreading process for various settings of parameters *β* and *γ*, examining the cumulative number of infected nodes per step of the process (see Supplementary Note 5 for more details about this experiment and Supplementary Fig. 4 for the results). We observed that, as the recovery probability *γ* decreases, the number of infected nodes increases both during the first time steps of the process, as well as at the end of the epidemic. This behavior is expected as high recovery rate *γ* implies that most of the nodes will move to the *R* state of the SIR model–thus being inactive in subsequent iterations of the model. Regarding the relative performance of the methods, we observed that it is not affected by the value of *γ*; the proposed **truss** method outperforms both baselines for all different settings of parameter *γ*. In the second case where we retain the recovery rate *γ* constant while the infection probability is increasing, we observed that the number of infected nodes increases. However, for higher values of *β*, the total number of infected nodes is almost the same for all methods. This behavior is rather expected; by increasing the infection rate, the importance of individual nodes in the epidemic process is reduced. For these values of *β*, the difference between the methods can be observed during the outbreak of the epidemic (i.e., first steps of the process), where the **truss** method performs qualitatively better.

## Discussion

Understanding and controlling the mechanisms that govern spreading processes in complex networks is a fundamental task in various domains, including disease propagation and viral marketing. Central to these tasks is the problem of identification of influential nodes with good spreading properties, that are able to diffuse information to a large part of the network. It has been empirically observed that widely used node centrality criteria such as degree and betweenness, have drawbacks when applied to find nodes with good spreading properties; a node may have a large number of neighbors but if it is located to the periphery of the network, its spreading capability is reduced. Kitsak *et al*.^15^ applied the *k*-core decomposition method in order to locate centrally placed individuals with good spreading properties; their observations suggested that the identified nodes outperform previously used criteria with respect to the spreading effectiveness. However, the main drawback of the *k*-core decomposition is that its resolution is quite coarse. Depending on the structure of the network, many nodes will be assigned the same *k*-core number, even if their spreading capability differs from each other (see also the results presented in Table 1 regarding the number of nodes of the maximal *k*-core subgraph of several real networks).

The fact that a relatively large fraction of the nodes that are extracted by the *k*-core decomposition method corresponds to highly influential nodes, was the motivating force behind our approach. To deal with this issue, we have considered the *K*-truss decomposition of a network–a triangle-based extension of the *k*-core structure. By setting a more strict criterion upon which nodes are assigned into layers of the graph, we have shown that the *K*-truss decomposition can effectively reduce the number of candidate influential spreaders in the network, as it further refines the set of nodes belonging to the maximal *k*-core subgraph (recall that the maximal *K*-truss is a subgraph of the maximal *k*-core). Using the SIR epidemic model, we have shown that such spreaders have the ability to influence a greater part of the network during the first steps of the process; also the total fraction of influenced nodes at the end of the epidemic is higher, compared to the performance of the rest nodes that belong to the maximal *k*-core subgraph and the top degree nodes of the network. Our experimental results also indicate that the *K*-truss decomposition filters out the best spreaders of the *k*-core structure; the spreading effectiveness of the remaining nodes is weakened, and those nodes show even worst behavior compared to the top degree ones (as indicated by the comparison of the **core** method to the **top degree**).

To further examine the spreading performance of the nodes located by the *K*-truss decomposition method, we studied the spreading achieved by each node in the graph. After ranking the nodes of the network with respect to their spreading effectiveness, we observed that those belonging to the maximal *K*-truss subgraph are distributed well among the optimal spreaders of the graph, presenting better behavior compared to the remaining nodes of the maximal *k*-core subgraph. Furthermore, we observed that the truss number in general, is closely related to the spreading effect. The nodes of the network are distributed among the optimal spreaders (after ranking) in a way that a relationship to truss number occurs.

An important issue about the *K*-truss decomposition method, is the computational complexity; it can be proportional to 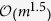, where 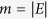 is the number of edges of the graph, since it requires the computation of the number of triangles that each node participates to. This is actually the main weak point of this method, compared to the widely used *k*-core decomposition of linear time complexity 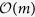. However, in this work, we are mainly interested in the nodes that belong to the maximal *K*-truss subgraph. By taking into account the fact that a *K*-truss subgraph is contained within a 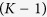-core subgraph, we can speedup the computation by firstly reducing the graph to its maximal core in linear time and then performing further refinements to extract the *K*-truss subgraph^26^.

It is worth noticing that most of the extensions presented for the *k*-core decomposition-based approach of Kitsak *et al*.^15^, can also be applied to our method (see Supplementary Note 6 for a description of some of these methods). One such case concerns the identification of multiple initial nodes, as our method is designed to detect single influential spreaders (this is the case of the influence maximization problem, where we should locate multiple initial seed nodes that are able to maximize the total spread of influence)^33^,^34^,^35^,^36^. The naive solution of choosing multiple nodes from the maximal *K*-truss subgraph will not perform well, since those nodes are clustered together in the graph and share many common neighborhood nodes. Thus, as suggested in the related literature^15^, a good strategy is to also consider the distance between them, as expressed by the number of hops needed to reach each other.

So far we have studied the effect of our method in real datasets by simulating spreading cascades. It is of great interest to also consider the identification of influential spreaders by following real information flow in social networks, as has been suggested by Pei *et al*.^37^. Unfortunately, a lot of difficulties arise in such a case considering the lack of ground truth information that can actually represent the diffusion of a specific idea as is simulated by epidemic models. Additionally, the problem of how to consider a time frame to analyze the influence of every node of the network arises, which can alter the results depending on the setting chosen. Finally, in real information flow, some nodes are not found performing any activity, making the comparison of the methods even harder. We have tested our method at the Facebook dataset^38^ and the nodes found after performing *K*-truss decomposition tend to be more effective in terms of spreading compared to those located by the *k*-core decomposition.

## Methods

### *k*-core decomposition

Let 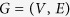 be an undirected graph with 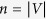 nodes and 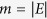 edges and let *H* be a subgraph of *G*, i.e., 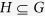. Subgraph *H* is defined to be a *k*-core subgraph of *G*, denoted by 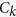, if it is a maximal connected subgraph in which all nodes have degree at least *k*. Then, each node 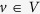 has a core number 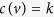, if it belongs to a *k*-core but not to a 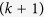-core. We denote as 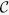 the set of nodes with the maximum core number 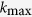 (i.e., the nodes of the *k*-core subgraph of *G* that corresponds to the maximum value of *k*)^16^. It is evident that if all the nodes of the graph have degree at least one, i.e., 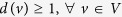, then the 1-core subgraph corresponds to the whole graph, i.e., 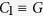. Furthermore, assuming that 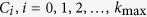 is the *i*-core of *G*, then the *k*-core subgraphs are nested, i.e., 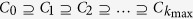.

Computing the *k*-core decomposition of a graph can be done through a simple process that is based on the following property: to extract the *k*-core subgraph, all nodes with degree less than *k* and their adjacent edges should be recursively deleted^16^. That way, beginning with 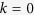, the algorithm removes all the nodes (and the incident edges) with degree equal or less than *k*, until no such nodes have been remained in the graph. Also notice that, removing edges that are incident to a node may cause reductions to the degree of neighboring nodes; the degree of some nodes may become at most *k*, and thus, they should also be removed at this step of the algorithm. When all remaining nodes have degree 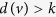, *k* is increased by one and the process is repeated until no more remaining nodes are left in the graph. Since each node and edge is removed exactly once, the running time of the algorithm is 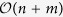
39. Batagelj and Zaveršnik later proposed an 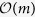 algorithm for *k*-core decomposition^17^.

### *K*-truss decomposition

The *K*-truss decomposition extends the notion of *k*-core using triangles, i.e., cycle subgraphs of length 3^26^,^27^. Let 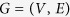 be an undirected graph. We define as a triangle 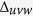 a cycle subgraph of nodes 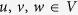. Additionally, the set of triangles of *G* is denoted by 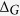. The support of an edge 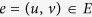 is defined as 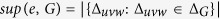 and expresses the number of triangles that contain edge *e*. Then, the *K*-truss, 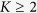, denoted by 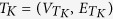, is defined as the largest subgraph of *G*, where every edge is contained in at least 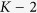 triangles within the subgraph, i.e., 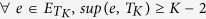. Respectively, the truss number of an edge 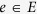 is defined as 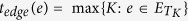. Thus, if 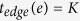, then the edge belongs to 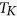 but not to 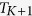, i.e., 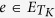 but 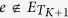. We use 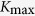 to denote the maximum truss number of any edge 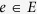. Since the definition of *K*-truss is per edge, we define as truss number of a node 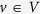, denoted by 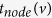, the maximum truss number of its incident edges, i.e., 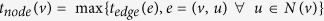, where 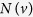 is the set of neighborhood nodes of *v*. We denote as 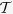 the set of nodes with maximum node truss number (in other words, this set contains the nodes of the maximal *K*-truss subgraph). The *K*-class of a graph 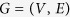 is defined as 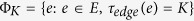. Then, the *K*-truss decomposition is defined as the task of finding the *K*-truss subgraphs of *G*, for all 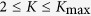. That is, the *K*-truss can be obtained by the union of all edges that have truss number at least *K*, i.e., 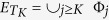.

The computation of the *K*-truss subgraph, for a specific value of 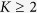, follows similar methodological procedure as the one of *k*-core, where instead of the degree of a node, we examine the number of triangles that the node participates to: remove all edges 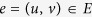 if they do not participate to at least 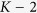 triangles, i.e., 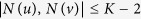. The time complexity of the method is 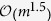 and the space complexity 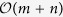. However, as we described in the Discussion section, here we are mostly interested to extract the nodes belonging to the maximal *K*-truss subgraph. This can be done effectively due to the fact that the maximal *K*-truss is a subgraph of the maximal *k*-core of the graph^26^.

### The SIR spreading model

To determine the spreading effect of specific nodes in the network, we apply the Susceptible-Infected-Revovered (SIR) epidemic model^30^,^31^,^40^. The model assumes a population of *N* individuals, divided on the following three states. Susceptible (S): the individual is not yet infected, thus being susceptible to the epidemic; Infected (I): the individual has been infected with the disease and it is capable of spreading the disease to the susceptible population; Recovered (R): after an individual has experienced the infectious period, it is considered as removed from the disease and it is not able to be infected again or to transmit the disease to others (immune to further infection or death).

Initially, all the nodes of the network are set at the susceptible state *S*, except from the one that we are interested to examine its spreading performance which is set at the infected state *I*. Then, at each time step *t* of the process, every node that is on the *I* state can infect its susceptible neighbors with probability *β* (called infection rate) and afterwards it can recover with probability *γ* (called recovery rate). Note that, a node cannot directly pass from state *I* to state *R* during the same time step *t* of the process.

## Additional Information

**How to cite this article**: Malliaros, F. D. *et al*. Locating influential nodes in complex networks. *Sci. Rep.* **6**, 19307; doi: 10.1038/srep19307 (2016).

## Supplementary Material

Supplementary Information

## Figures and Tables

**Table 1:** Properties of the real-world graphs used in this study.

Network Name	Nodes	Edges	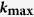	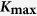	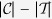	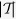	*τ*
Email-Enron	33,696	180,811	43	22	230	45	0.00840
Epinions	75,877	405,739	67	33	425	61	0.00540
Wiki-Vote	7,066	100,736	53	23	286	50	0.00720
Email-EuAll	224,832	340,795	37	20	230	62	0.00970
Slashdot	82,168	582,533	55	36	38	96	0.00074
Wiki-Talk	2,388,953	4,656,682	131	53	463	237	0.00870

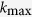
 and 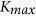 denote the maximum *k*-core and *K*-truss numbers respectively (as producedC by the decompositions); 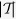 represents the number of nodes belonging to set 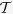; 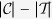 represents the number of the nodes belonging to set 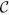 (i.e., the nodes of the maximal *k*-core subgraph), excluding the nodes that belong to set 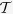; *τ* is the epidemic threshold of the graph and is defined to be equal to 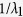, where 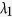 is the largest eigenvalue of the adjacency matrix of the network.

**Table 2:** Evaluation of the spreading performance per step of the process.

		Time Steps							
	Method	2	4	6	8	10	*Final step*	*σ*	*Max step*
Email-Enron	truss	8.44	46.66	204.08	418.77	355.84	2,596.52	136.7	33
	core	4.78	31.97	152.55	367.28	364.13	2,465.60	199.6	37
	top degree	6.89	34.13	155.48	360.89	357.08	2,471.67	354.8	36
Epinions	truss	4.17	19.70	75.04	204.14	329.08	2,567.69	227.8	37
	core	3.45	14.72	55.27	158.56	280.03	2,325.37	327.2	43
	top degree	4.22	16.03	58.84	166.23	289.49	2,414.99	331.7	47
Wiki-Vote	truss	2.92	6.92	15.27	28.73	42.46	560.66	114.9	52
	core	1.92	4.78	10.65	20.66	32.40	466.01	104.5	57
	top degree	2.43	5.46	12.05	23.05	35.55	502.88	104.5	62
Email-EuAll	truss	11.62	62.25	240.97	584.87	725.42	5,018.52	487.94	36
	core	9.85	40.82	158.72	433.81	644.76	4,579.84	498.71	38
	top degree	17.96	39.93	144.69	503.18	548.25	4,137.56	1,174.84	39
Slashdot	truss	5.36	66.21	461.35	1,390.52	1,359.99	8,207.46	368.37	32
	core	6.48	61.13	410.19	1,272.29	1,344.33	8,002.76	518.43	32
	topdegree	13.95	83.29	483.95	1,426.81	1,403.80	8,489.45	59.01	32
Wiki-Talk	truss	64.21	3,259.05	34,543.23	9,853.84	1,186.41	93,491.81	476.22	21
	core	41.77	2,027.69	31,223.21	13,055.45	1,664.52	93,496.50	767.35	23
	top degree	88.84	2,475.01	29,694.45	13,720.15	1,937.89	93,411.18	1,166.77	24

Average number of infected nodes per step of the SIR model for the proposed **truss** method and the two baselines **core** and **top degree**, using *β* close to the epidemic threshold of each graph and *γ* = 0.8. At the *Final step* column, we show the total number of infected nodes at the end of the process (*Max step*), with standard deviation *σ*. Observe that, in almost all datasets the **truss** method achieves higher spreading especially during the first steps of the process. Also, as the *Max step* column indicates, the epidemic dies out at an earlier time step when triggered by a node of the maximal *K*-truss subgraph.
