# Supplementary material for: Locating influential nodes in complex networks
Source: Sci Rep. 2016 Jan 18;6:19307. doi: 10.1038/srep19307 (PMC4725982; doi:10.1038/srep19307)
Supplement: Supplementary Information [file srep19307-s1.pdf]

LOCATING INFLUENTIAL NODES IN COMPLEX NETWORKS

Supplementary Information

FRAGKISKOS D. MALLIAROS, MARIA-EVGENIA G. ROSSI, MICHALIS VAZIRGIANNIS

Computer Science Laboratory, École Polytechnque, 91120 Palaiseau, France

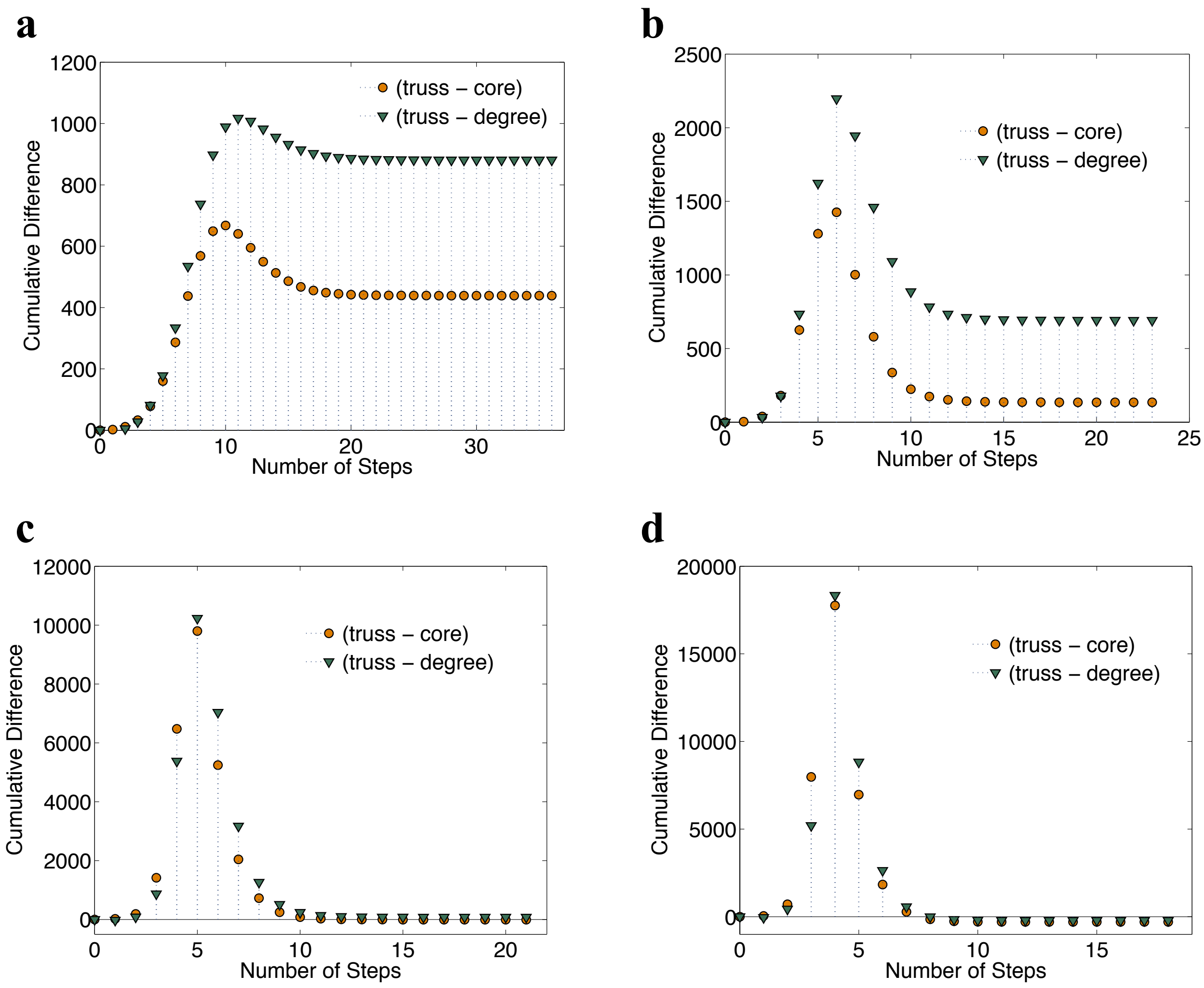

SUPPLEMENTARY FIGURE 1. **Comparative performance of the proposed truss method versus the core and top degree methods.** This figure complements Figure 1 of the main text. We show results for the following networks and the corresponding infection rates β: EMAIL-EUALL **(a)** $\beta = 0.01$, **(b)** $\beta = 0.03$; WIKI-TALK **(c)** $\beta = 0.01$, **(d)** $\beta = 0.03$. Each plot depicts the cumulative difference of the infected nodes per step achieved by the **truss** method vs. the **core** (truss - core) and **top degree** (truss - degree) methods. Parameter γ of the SIR models is set to $\gamma = 0.8$. In all cases, the proposed **truss** method outperforms the baselines, leading to more effective information spreading.

		Time Step											
	**Method**	2	3	4	5	6	7	8	9	10	*Final step*	σ	*Max step*
EMAIL-ENRON	**truss**	8.44	18.58	46.66	104.11	204.08	328.39	418.77	425.06	355.84	2, 596.52	136.7	33
	**core**	4.78	12.82	31.97	73.77	152.55	264.36	367.28	403.98	364.13	2, 465.60	199.6	37
	**top degree**	6.89	13.87	34.13	76.67	155.48	264.13	360.89	394.37	357.08	2, 471.67	354.8	36
EPINIONS	**truss**	4.17	9.25	19.70	39.56	75.04	130.48	204.14	278.69	329.08	2, 567.69	227.8	37
	**core**	3.45	7.18	14.72	29.11	55.27	98.11	158.56	226.17	280.03	2, 325.37	327.2	43
	**top degree**	4.22	7.94	16.03	31.32	58.84	103.91	166.23	234.96	289.49	2, 414.99	331.7	47
WIKI-VOTE	**truss**	2.92	4.37	6.92	10.43	15.27	21.63	28.73	35.93	42.46	560.66	114.9	52
	**core**	1.92	3.07	4.78	7.22	10.65	15.18	20.66	26.70	32.40	466.01	104.5	57
	**top degree**	2.43	3.53	5.46	8.17	12.05	17.04	23.05	29.49	35.55	502.88	104.5	62
EMAIL-EUALL	**truss**	11.62	28.04	62.25	127.79	240.97	405.53	584.87	705.89	725.42	5,018.52	487.94	36
	**core**	9.85	18.69	40.82	82.28	158.72	279.41	433.81	574.97	644.76	4,579.84	498.71	38
	**top degree**	17.96	16.74	39.93	73.66	144.69	384.07	503.18	565.06	548.25	4,137.56	1, 174.84	39
SLASHDOT	**truss**	5.36	20.57	66.21	188.52	461.35	917.2	1,390.52	1,571.97	1,359.99	8,207.46	368.37	32
	**core**	6.48	19.68	61.13	168.36	410.19	820.77	1,272.29	1,486.5	1,344.33	8,002.76	518.43	32
	**top degree**	13.95	27.88	83.29	204.60	483.95	940.49	1,426.81	1,616.55	1,403.80	8,489.45	59.01	32
WIKI-TALK	**truss**	64.21	435.79	3,259.05	16,227.25	34,543.23	23,818.06	9,853.84	3,487.65	1,186.41	93,491.81	476.22	21
	**core**	41.77	269.96	2,027.69	11,169.2	31,223.21	28,732.06	13,055.45	4,805.11	1,664.52	93,496.50	767.35	23
	**top degree**	88.84	324.11	2,475.01	11,718.28	29,694.45	27,009.05	13,720.15	5,396.45	1,937.89	93,411.18	1, 166.77	24

SUPPLEMENTARY TABLE 1. **Evaluation of the spreading performance per step of the process.** This table is an extended version of Table 2 of the main text. Average number of infected nodes per step of the SIR model for the proposed **truss** method and the two baselines **core** and **top degree**, using β close to the epidemic threshold of each graph and $\gamma = 0.8$. At the *Final step* column, we show the total number of infected nodes at the end of the process (*Max step*), with standard deviation σ. Observe that, in almost all datasets the **truss** method achieves higher spreading especially during the first steps of the process. Also, as the *Max step* column indicates, the epidemic dies out at an earlier time step when triggered by a node of the maximal K-truss subgraph.

		Time Step										
	**Method**	2	3	4	5	6	7	8	9	10	*Final step*	σ	*Max step*
EMAIL-ENRON	**truss**	9.44	28.03	74.69	178.80	382.88	711.27	1,130.05	1,555.11	1,910.95	2,596.52	136.7	33
	**core**	5.78	18.60	50.57	124.35	276.90	541.26	908.54	1,312.52	1,676.65	2,465.60	199.6	37
	**top degree**	7.89	21.76	55.90	132.57	288.05	552.18	913.07	1,307.45	1,664.53	2,471.67	354.8	36
EPINIONS	**truss**	5.17	14.42	34.13	73.69	148.74	279.23	483.37	762.06	1,091.14	2,567.69	227.8	37
	**core**	4.45	11.64	26.36	55.48	110.75	208.87	367.43	593.59	873.62	2,325.37	327.2	43
	**top degree**	5.22	13.16	29.20	60.52	119.36	223.27	389.49	624.46	913.95	2,414.99	331.7	47
WIKI-VOTE	**truss**	3.92	8.30	15.23	25.66	40.94	62.57	91.31	127.25	169.71	560.66	114.9	52
	**core**	2.92	5.99	10.78	18.01	28.66	43.85	64.50	91.20	123.60	466.01	104.5	57
	**top degree**	3.43	6.96	12.43	20.61	32.66	49.70	72.75	102.25	137.81	502.88	104.5	62
EMAIL-EUALL	**truss**	12.62	40.66	102.92	203.72	471.69	877.22	1,462.10	2,168.00	2,893.43	5,018.52	487.94	36
	**core**	10.85	29.55	70.37	152.65	311.38	590.79	1,024.60	1,599.57	2,244.34	4,579.84	498.71	38
	**top degree**	18.96	35.71	75.64	149.30	294.00	543.45	927.52	1,430.70	1,995.77	4,137.56	1,174.84	39
SLASHDOT	**truss**	6.36	26.93	93.14	281.67	743.03	1,660.23	3,050.75	4,622.73	5,982.73	8,207.46	368.37	32
	**core**	7.48	27.17	88.31	256.67	666.86	1,487.64	2,759.93	4,246.43	5,590.76	8,002.76	518.43	32
	**top degree**	14.95	42.84	126.13	330.74	814.69	1,755.18	3,181.99	4,798.55	6,202.35	8,489.45	59.01	32
WIKI-TALK	**truss**	65.21	501.00	3,760.06	19,987.31	54,530.55	78,348.62	88,202.46	91,690.11	92,876.53	93,491.81	476.22	21
	**core**	42.77	312.74	2,340.43	13,509.64	44,732.85	73,104.92	86,160.38	90,965.49	92,630.01	93,496.5	767.35	23
	**top degree**	89.84	413.95	2,888.96	14,607.24	44,301.69	71,310.74	85,030.90	90,427.35	92,365.25	93,411.18	1,166.77	24

SUPPLEMENTARY TABLE 2. **Evaluation of the cumulative spreading performance.** Cumulative number of infected nodes per step of the SIR model for the proposed **truss** method and the two baselines **core** and **top degree**, using β close to the epidemic threshold of each graph and $\gamma = 0.8$. At the *Final step* column, we show the total number of infected nodes at the end of the process (*Max step*), with standard deviation σ. Observe that, in almost all datasets the **truss** method achieves higher cumulative number of infected nodes.

SUPPLEMENTARY NOTE 1: DESCRIPTION OF THE DATASETS

In this work we have mainly experimented with social networks, but our results can also be extended to networks of other disciplines. A full description of the datasets used in the paper follows, while some basic properties are demonstrated in Table 1 of the main text. All datasets are publicly available in [1].

(i) EMAIL-ENRON. This email communication network created by email interaction between the members of Enron Corporation, and made public by the Federal Energy Regulatory Commission during its investigation [2]. It covers data from 150 users and a total of half a million messages. Each node represents an email address and an undirected edge was formed between two nodes if at least an address i sent an email to address j.

(ii) EMAIL-EUALL. This email network was collected from email communication of a large research institution [3]. To create the graph, each email address is considered as a node and an edge is created between two nodes if the latter have exchanged messages both ways. Overall there are $3,038,531$ emails between $287,755$ different email addresses recorded from October 2003 to May 2005.

(iii) EPINIONS. This is a trust-based (who-trusts-whom) online social network between the members of the Epinions.com (`www.epinions.com`) product review website [4]. The nodes of the network correspond to users of the website and the edges capture trust relationships between them. Although the network is signed, in our experiments we discard this information; we also convert the graph to an undirected one to use it on our experiments.

(iv) WIKI-VOTE. The graph was created from the online encyclopedia Wikipedia (`www.wikipedia.org`) and more precisely from the elections conducted to promote users to administrators (till January 2008) [5]. The nodes of the social network correspond to Wikipedia users and an edge between users i, j denotes that user i voted for user j.

(v) WIKI-TALK. This network is also created by data imported from Wikipedia [5]. Each user has a talk page where all interested users can edit to update various articles on Wikipedia. The specific dataset contains all discussions between users until January 2008. The nodes of this network correspond to users of Wikipedia and an edge from node i to node j indicates that user i edited a talk page of user j at least once.

(vi) SLASHDOT. Slashdot (`slashdot.org`) is a technology news website. The nodes of the social network that is created correspond to users and the edges capture friendship relationships among them (till February 2009) [6]. In fact, users are able to tag other users as friends or foes, forming a signed social network with positive and negative types of edges. In our experiments, we do not take into account the type of the edges.

Supplementary Note 2: Properties of the K-truss Subgraphs

We have examined the distribution of the node truss numbers t_{node} of the graphs presented in Table 1 of the main text and the results are depicted in Supplementary Fig. 2. Each plot shows the complementary cumulative distribution function (CCDF) of the nodes' truss number in log-log scale. As we can observe, in most of the cases the distribution is skewed, indicating that very few nodes have high truss number; the majority of the nodes belong to "low" K-truss subgraphs, i.e., small values of parameter K of the decomposition. We have also fitted a power-law distribution [7] to the data (red colored line) and the exponent is shown in Supplementary Fig. 2. Here, we do not claim that the truss number distribution is fully captured by a power-law; nevertheless, it corresponds to heavy-tailed distribution and this fact can help us to better understand the underlying properties of the data. In our case, this means that we can reduce the graph into a subgraph with exponentially smaller size and try to locate influential spreaders within this subgraph.

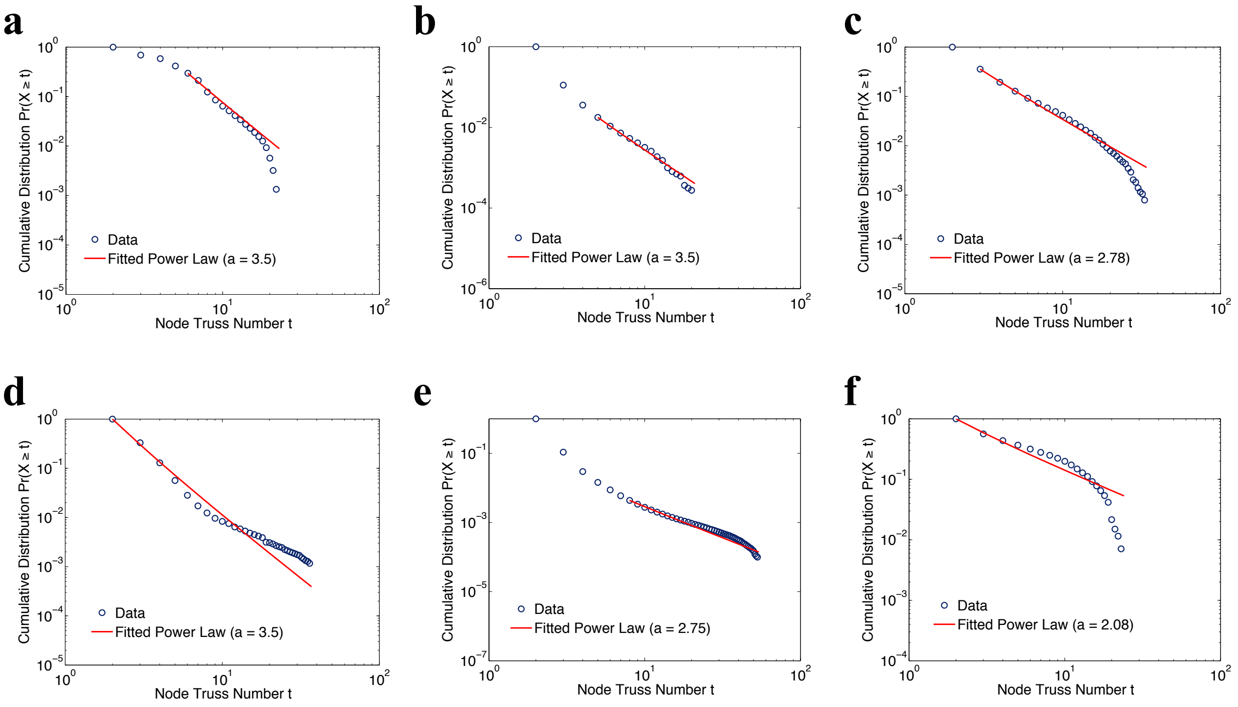

Supplementary Figure 2. **Complementary cumulative truss number distribution function.** **(a)** Email-Enron; **(b)** Email-EuAll; **(c)** Epinions; **(d)** Slashdot; **(e)** Wiki-Talk; **(f)** Wiki-Vote; Each plot depicts the distribution of the truss numbers for the nodes of the graph on log-log scale. The red line corresponds to the fitted power-law distribution.

Supplementary Note 3: Comparison of Maximal K-truss and k-core Subgraphs

It has been shown that the maximal k-core and K-truss subgraphs (i.e., maximum values for k, K) overlap, with the latter being a subgraph of the former; in fact, K-truss represents the core of a k-core that filters out less important information. Supplementary Fig. 3 shows an example of a graph and its k-core and K-truss subgraphs respectively. The red colored nodes correspond to the set $\mathcal{C}$ (i.e., the maximal k-core subgraph of the graph). The edges of the gray shadowed region are the edges that belong to the maximal K-truss of the original graph and the corresponding nodes are those belonging to set $\mathcal{T}$, i.e., the nodes with the maximum node truss number. In our approach, we argue that those nodes correspond to highly influential ones in the graph.

As we discussed in the main text, the K-truss decomposition is computationally a more difficult task compared to the one of k-core decomposition. In our approach, as we are only interested for the maximal K-truss subgraph, we take into account the structure shown in Supplementary Fig. 3. That is, we first compute the maximal k-core subgraph in linear time with respect to the total number of edges (subgraph defined by the red colored nodes in Supplementary Fig. 3) and then we extract the maximal K-truss subgraph (gray area in Supplementary Fig. 3). That way, the overall complexity of the task is significantly reduced.

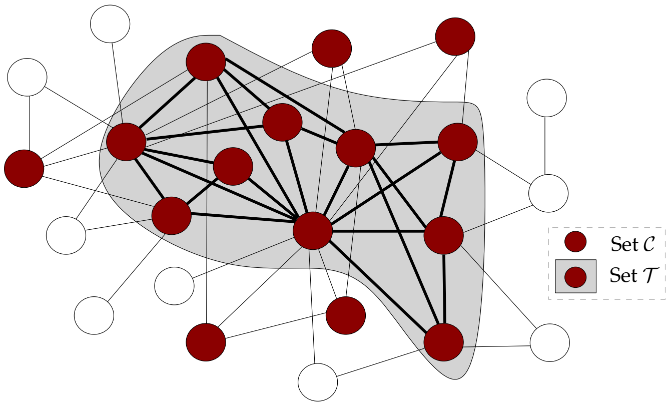

Supplementary Figure 3. **Schematic representation of the maximal k-core and K-truss subgraphs of a graph.** The red colored nodes correspond to the 3-core subgraph of the graph (set $\mathcal{C}$); the gray shadowed region indicate the 4-truss subgraph (set $\mathcal{T}$).

Supplementary Note 4: Simulating the Spreading Process

Supplementary Algorithm 1 presents the steps of the proposed framework for (i) selecting the initial node that will trigger the epidemic (cascade) and (ii) evaluate the impact of this individual node with respect to the epidemic spreading under the SIR model. Initially, we choose a node that belongs to $\mathcal{T}$ (i.e., maximal K-truss subgraph) and set it to the infected (I) state. Notice that, we keep track of the infected, susceptible and recovered nodes for each time step of the process. At each time step, an infected node can infect a susceptible neighbor with probability β. Additionally, any node that got infected at previous time steps of the process, can recover with probability γ. The process is repeated until no more infected nodes left. Finally, the algorithm returns M_v which is the number of the infected individuals under the cascade triggered by node v.

Supplementary Algorithm 1 Identify nodes and evaluate spreading process

---

Input: Undirected graph $G = (V, E)$, parameters β, γ
Output: Size of infected population M_v for cascade triggered by node v

Select node $v \in \mathcal{T}$
$State(v) \leftarrow I$, $State(V \setminus v) \leftarrow S$ /* Initialization steps */
$I(0) \leftarrow \{v\}$, $S(0) \leftarrow V \setminus v$, $R(0) \leftarrow \emptyset$
$t \leftarrow 0$
repeat
    $t \leftarrow t + 1$
    $I(t) \leftarrow \emptyset$, $R(t) \leftarrow \emptyset$
    for each node $w \in V$ **do**
        /* Infected (I) nodes can infect susceptible neighbors */
        if $State(w) = I$ **then**
            for each node $z \in \{Nb(w) : State(z) = S\}$ **do**
                $\Pr(State(z) \leftarrow I) = \beta$ (also $I(t) \leftarrow I(t) \cup \{z\}$)
            end for
        end if
        /* Nodes that got infected at previous time steps can recover (R) */
        if $State(w) = I$ and $w \notin I(t)$ **then**
            $\Pr(State(w) \leftarrow R) = \gamma$ (also $R(t) \leftarrow R(t) \cup \{w\}$)
        end if
    end for
until $I(t) = \emptyset$ /* No more infected nodes left */
return $M_v \leftarrow I(1) \cup I(2) \cup \ldots \cup I(t)$

Supplementary Note 5: Impact of Infection and Recovery Rate on the Spreading Process

As we presented in the main text, parameters β and γ of the SIR model have been set to some constant values; the infection rate β is typically set close to the epidemic threshold of the graph (as defined by the maximum eigenvalue of the adjacency matrix of the graph), while the recovery rate is considered constant and always set to $\gamma = 0.8$. Here, we examine the impact of the infection and recovery rate on the epidemic spreading achieved by the proposed method (**truss**) and the two baseline methods (**core** and **top degree**). To that end, we simulate the spreading process for each of the above methods, setting parameters β and γ as follows:

(i) Parameter β is set close to the epidemic threshold of the graph, while varying parameter $\gamma \in \{0.5, 0.8, 1\}$. Parameter $\gamma = 1$ implies that each infected node moves to the recovered (R) state with probability one, in the next step of the model.

(ii) The recovery rate is set to $\gamma = 0.8$, while considering different values of parameter β, always above the epidemic threshold of the graph. As we discussed in the main text, if we consider high values of the infection rate β, a relatively high fraction of nodes will be infected, and thus, the spreading capabilities of individual nodes is diminished.

Supplementary Fig. 4 shows the results. In all cases, we have computed the cumulative fraction of infected nodes I_t per step of the process, for each of the three methods, along with the standard deviation (depicted as error bars in the plot). As we can observe, while the recovery probability γ decreases, the number of infected nodes increases both during the first time steps of the process, as well as at the end of the epidemic. This behavior is expected since, as we discussed above, with high recovery rate γ most of the nodes will move to the R state, thus being inactive in subsequent iterations of the model. Regarding the performance of the methods, it is evident that the proposed **truss** outperforms both baselines for all different settings of parameter γ.

In the second case where the recovery rate γ is constant, while the infection probability is increasing, the number of infected nodes naturally increases. However, for higher values of β, the total number of infected nodes is almost the same for all methods. This behavior is rather expected; by increasing the infection rate, the importance of individual nodes in the epidemic process is reduced. For these values of β, the difference between the methods can be observed during the outbreak of the epidemic (i.e., first steps of the process), where the **truss** method performs qualitatively better.

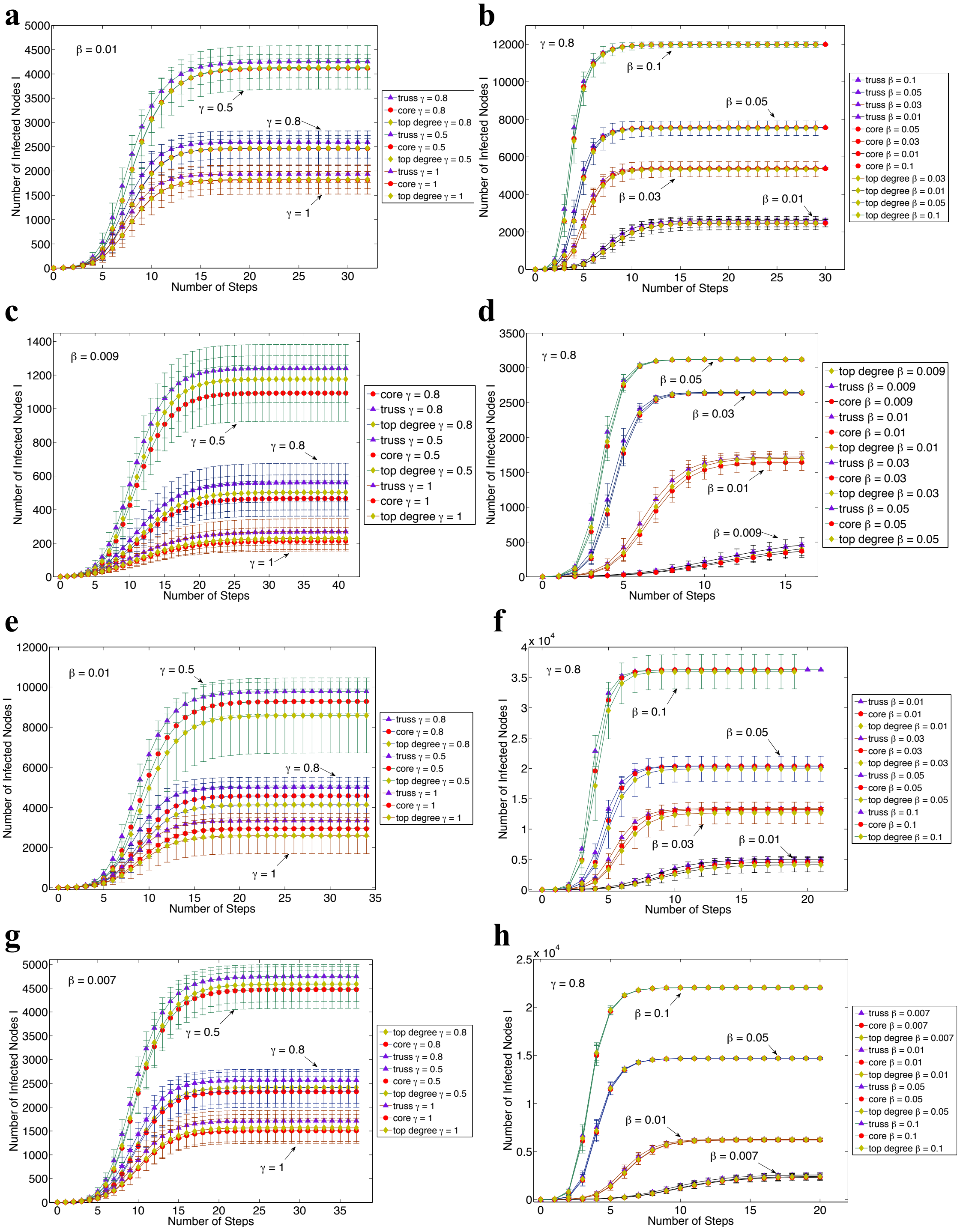

SUPPLEMENTARY FIGURE 4. **Impact of infection and recovery probabilities of the SIR model on the spreading process**. EMAIL-ENRON: **(a)** $\beta = 0.01$, **(b)** $\gamma = 0.8$; WIKI-VOTE: **(c)** $\beta = 0.009$, **(d)** $\gamma = 0.8$; EMAIL-EUALL: **(e)** $\beta = 0.001$, **(f)** $\gamma = 0.8$; EPINIONS: **(g)** $\beta = 0.007$, **(h)** $\gamma = 0.8$. We have examined the following settings: (i) parameter β is set close to the epidemic threshold of the graph, while varying parameter $\gamma \in \{0.5, 0.8, 1\}$ (left column); (ii) set parameter $\gamma = 0.8$ and consider different values of parameter β always above the epidemic threshold of the graph (right column).

SUPPLEMENTARY NOTE 6: EXTENDED RELATED WORK

In this section, we briefly discuss extended related work for the problem of identification of influential spreaders. Building upon the fact that the k-core decomposition is an effective (and efficient) measure to capture the spreading properties of nodes, as introduced by Kitsak et al. [8], several extensions have been proposed. The authors of Supplementary Ref. [9] introduced a modified version of the k-core decomposition in which the nodes are ranked taking into account their connections to the remaining nodes of the graph as well as to the removed nodes at previous steps of the process. They showed that the proposed node ranking method is able to identify nodes with better spreading properties compared to the traditional k-core decomposition. Bae et al. [10] extended the metric of k-core number of each node by considering the core number of its neighbors. That way, the ranking produced by the method is more fine-grained in the sense that the effect of assigning the same score (i.e., k-core number) to many nodes is eliminated. Basaras et al. [11] proposed to rank the nodes according to a criterion that combines the degree and the k-core number of a node within an μ-hop neighborhood. In Supplementary Ref. [12] the authors introduced a criterion that combines three previously examined measures, namely degree, betweenness centrality and core number. The intuition was that, most of the widely used centrality criteria produce highly correlated rankings of nodes; combining them in a proper way, we are able to achieve a more accurate indicator of influential nodes. Zhang et al. [13] proposed a method to locate influential nodes taking into account the existence of community structure in networks. In Supplementary Ref. [14], the authors considered real social media data, in order to examine to what extend the structural position of a user in the network allows us to characterize the ability of an individual to spread rumors effectively. Their results indicate that although the most appropriate feature is the degree of a node, only a few such highly-connected individuals exist; however, by considering the k-core number metric, we are able to locate a larger set of individuals that are likely to trigger large cascades. For a detailed review in the area, we refer to the article by Pei and Makse [15]. As we also discussed in the main text, it is important to stress out that most of the above mentioned extensions can also be applied to the proposed K-truss decomposition-based approach.

SUPPLEMENTARY REFERENCES

[1] Jure Leskovec and Andrej Krevl. SNAP Datasets: Stanford large network dataset collection. `http://snap.stanford.edu/data`, June 2014.

[2] Klimt, Bryan and Yang, Yiming. The enron corpus: A new dataset for email classification research *Machine learning: ECML 2004*,217–226, 2004. Springer Berlin Heidelberg.

[3] Leskovec, Jure, Jon Kleinberg, and Christos Faloutsos. Graph evolution: Densification and shrinking diameters. *ACM Transactions on Knowledge Discovery from Data (TKDD) 1*, no. 1 (2007): 2

[4] Richardson, Matthew and Agrawal, Rakesh and Domingos, Pedro. Trust management for the semantic web *The Semantic Web-ISWC 2003*,351–368, 2003. Springer Berlin Heidelberg.

[5] Leskovec, Jure and Huttenlocher, Daniel and Kleinberg, Jon. Predicting positive and negative links in online social networks *Proceedings of the 19th international conference on World wide web*,641–650, 2010. ACM.

[6] Leskovec, Jure, Kevin J. Lang, Anirban Dasgupta, and Michael W. Mahoney. Community structure in large networks: Natural cluster sizes and the absence of large well-defined clusters. *Internet Mathematics 6*, no. 1 (2009): 29-123.

[7] Aaron Clauset, Cosma Rohilla Shalizi, and M. E. J. Newman. Power-law distributions in empirical data. *SIAM Rev.*, 51(4):661–703, 2009.

[8] Maksim Kitsak, Lazaros Gallos, Shlomo Havlin, Fredrik Liljeros, Lev Muchnik, H. Eugene Stanley, and Hermán Makse. Identification of Influential Spreaders in Complex Networks. *Nature Physics*, 6(11):888–893, Aug 2010.

[9] An Zeng and Cheng-Jun Zhang. Ranking spreaders by decomposing complex networks. *Physics Letters A*, 377(14):1031–1035, 2013.

[10] Joonhyun Bae and Sangwook Kim. Identifying and ranking influential spreaders in complex networks by neighborhood coreness. *Physica A: Statistical Mechanics and its Applications*, 395:549–559, 2014.

[11] Pavlos Basaras, Dimitrios Katsaros, and Leandros Tassiulas. Detecting influential spreaders in complex, dynamic networks. *Computer*, 46(4):24–29, 2013.

[12] Bonan Hou, Yiping Yao, and Dongsheng Liao. Identifying all-around nodes for spreading dynamics in complex networks. *Physica A: Statistical Mechanics and its Applications*, 391(15):4012–4017, 2012.

[13] Xiaohang Zhang, Ji Zhu, Qi Wang, and Han Zhao. Identifying influential nodes in complex networks with community structure. *Knowledge-Based Systems*, 42:74–84, 2013.

[14] Javier Borge-Holthoefer, Alejandro Rivero, and Yamir Moreno. Locating privileged spreaders on an online social network. *Phys. Rev. E*, 85:066123, 2012.

[15] Sen Pei and Hernn A Makse. Spreading dynamics in complex networks. *Journal of Statistical Mechanics: Theory and Experiment*, 2013(12):P12002, 2013.
